# Modeling to Optimize Terminal Stem Cell Differentiation

**DOI:** 10.1155/2013/574354

**Published:** 2013-02-11

**Authors:** G. Ian Gallicano

**Affiliations:** Department of Biochemistry and Molecular & Cellular Biology, Georgetown University Medical Center, Washington, DC 20057, USA

## Abstract

Embryonic stem cell (ESC), iPCs, and adult stem cells (ASCs) all are among the most promising potential treatments for heart failure, spinal cord injury, neurodegenerative diseases, and diabetes. However, considerable uncertainty in the production of ESC-derived terminally differentiated cell types has limited the efficiency of their development. To address this uncertainty, we and other investigators have begun to employ a comprehensive statistical model of ESC differentiation for determining the role of intracellular pathways (e.g., STAT3) in ESC differentiation and determination of germ layer fate. The approach discussed here applies the Baysian statistical model to cell/developmental biology combining traditional flow cytometry methodology and specific morphological observations with advanced statistical and probabilistic modeling and experimental design. The final result of this study is a unique tool and model that enhances the understanding of how and when specific cell fates are determined during differentiation. This model provides a guideline for increasing the production efficiency of therapeutically viable ESCs/iPSCs/ASC derived neurons or any other cell type and will eventually lead to advances in stem cell therapy.

## 1. Introduction

 Two decades of researching stem cells has brought us to a point where they are beginning to be used to treat or cure diseases. The “big three” stem cell types that comprise the heart of research include embryonic stem cells (ESCs), induced pluripotent stem cells (iPSCs), and adult stem cells (ASCs). Although the 1970s–1990s saw many exciting discoveries pertaining to mouse ESCs and various types of ASCs, the field lit up in the late 1990s with the discovery by James Thompson's group that human ESCs could be cultured *in vitro*. ESCs, which are derived from the inner cell mass (ICM) of blastocyst stage embryos, have the ability to undergo self-renewal and to differentiate into any of the three primary germ layers [1, 2]. This characteristic, that is, pluripotency, is the basis for the idea of stem cell therapies [3]. It is widely believed that by harnessing the power of ESC differentiation researchers can guide them into specific mature cell types that can be used to replace dead or damaged cells in various disease states. However, preliminary studies have shown limited success in producing large, pure populations of cells for use as potential therapies [4–8].

While ESCs, iPCs, and some ASCs are among the most promising treatments for heart failure, spinal cord injury, neurodegenerative diseases, and diabetes, considerable uncertainty in the production of ESC, iPSC, and ASC-derived terminally differentiated cells such as cardiomyocytes, *β*-islet cells, and dopaminergic neurons has limited the progress of their development and their use as therapies [10–12]. Research has shown that ESCs, iPCs, and ASCs consistently and reproducibly differentiate into many cell types; however, there are very little data to support the idea that fate commitment during differentiation follows the same temporal patterns found during development *in vivo*. As ESCs and iPSC differentiate, they form embryoid bodies (EBs) which loosely resemble blastocyst stage and egg-cylinder stage embryos. In contrast to embryos, EBs lack the organized structure of the developing embryo. Adding to this problem, ASCs are not normally induced to differentiate through EB protocols. Therefore, the inductive signals that regulate early differentiation events, such as formation of the primary germ layers, may not occur in ESC, iPSC, and ASC differentiation as expected when compared to embryogenesis. As a result, this incomplete understanding of the process of differentiation *in vitro* has led to the development of inefficient and somewhat haphazard protocols for inducing differentiation directed toward specific cell fates. For example, current techniques for stimulating neural differentiation generate terminally differentiated neural cells, which may or may not be capable of forming functional synapses when injected for therapeutic purposes. In addition, the EB method of differentiation results in populations of mixed cell types, including some undifferentiated cells, which, in the case of ESCs and iPSCs, increase the risk of teratoma formation upon injection. Thus, a more detailed understanding of ESC, iPSC, and ASC differentiation and specifically the time course of commitment to terminally differentiated fates is necessary to increase the potential success of stem cell based therapies. 

 In my laboratory, we have placed a great deal of focus on identifying the role of STAT3 in development and stem cell biology. Depending on the downstream inductive signals, STAT3 has been shown to play a major role in the processes of both ESC proliferation and differentiation into cardiac and neural cell types [13–20]. To date, the functional importance of the STAT3 pathway in cardiomyocyte differentiation has been more clearly defined than for neural lineages where STAT3, once thought to be primarily important for glial cell differentiation, has recently been shown also to be vital for ESC differentiation into neural stem cells (NSCs) [14]. In essence, the regulation of STAT3 activity may be a valid target in designing protocols to increase production of cardiomyocytes and ectodermal lineages including NS and neuronal cell types. 

 Based on the decades of work in the literature and our own results, it has been hypothesized that the process of ESC/iPSC/ASC differentiation is complex but consistent, allowing fate commitment to various cell lineages that can be measured over time, and be modeled by statistical methods. In fact, two questions have arisen that, if investigated and answered, could significantly contribute to the future success of stem cell therapies. First, is it possible to generate a comprehensive model of stem cell differentiation? Such a model would identify the probability of the formation of the primary germ layers, over the course of time, creating a tool that researchers could use in analyzing and designing ESC, iPSC, and/or ASC differentiation protocols. Second, how would one apply a statistical model for generating highly purified populations of desired cell types? For example, could it be possible to more clearly define roles for members of distinct signaling pathways, such as the Jak/STAT3 pathway, during differentiation of multipotent/pluripotent cells into specific lineages (i.e., ectoderm, endoderm, and mesoderm)? Here, I show that, upon revisiting a type of statistical modeling method using ESC differentiation as a baseline, it is possible to measure and identify the effect of manipulating a signaling mechanism. Here, I also describe the effects that a naturally occurring, dominant-negative form of STAT3 known as STAT3*β* have on the progression of ESC differentiation. Although analyses could be on either the cardiomyocyte or neural lineages or both, the focus for this paper will be placed on Stat3 within a model of neuroectodermal fate commitment. The approach could easily be applied to iPSCs and ASCs.

## 2. Overview of the Statistical Analyses 

To answer these two questions posed above, the techniques of choice for analyzing ESC differentiation usually include basic morphological observations and flow cytometry, which when used with ESC model systems can track expression changes of pluripotent to germ layer specific markers within individual cells over the time course of differentiation; however, the key to forming a reliable model to answer these questions is the method of data analysis. We and others have advanced this endeavor by creating a comprehensive probabilistic model of stem cell differentiation using the Bayesian methodology to frame an iterative overall experimental analysis. For example, a study can use this methodology to follow ESCs or iPSCs each day during differentiation with or without addition of any differentiation-inducing factors. Why is there focus on FACS analyses instead of other conventional techniques such as Western blots and/or qRT-PCR? Because EBs or ASCs are enmeshed in a heterogeneous populations, and RT-PCR and Western blot techniques, which analyze whole populations of cells, are not sufficient for studying differentiation within the milieu. Flow cytometry would enable analyses of varying numbers of individual cells that differentiate, regardless of cell fate, on each day of differentiation. Based on these data, curve fitting software is utilized to define a model of the time course of stem cell differentiation. Of course, the flow data is usually confirmed by qRT-PCR and Western analyses of the purified cell populations. The results gathered using all of these techniques are then used to formulate a predicted value for the peak day of differentiation (i.e., time when the slope of the curve is greatest).

The next step of this approach focuses on narrowing the the time course of differentiation in order to predict the value for the peak day of differentiation. During this set of experiments, the individual probabilities of differentiation into each of the three primary germ layers, on every day of the time course, are calculated. Probabilistic analyses are implemented to address the variability in the time course of differentiation within EBs. Graphing the probabilities of differentiation versus time would yield a visual tool identifying the time points at which the differentiation into each germ layer is at a maximum. Although it is the ectoderm that gives rise to neural tissue, differentiation is an inductive process, and formation of ectoderm relies on signals from both the endoderm and mesoderm. Therefore, differentiation of all three germ layers can be analyzed using this approach. 

 How could this model system be used as a tool for identifying specific pathways? Although there are many differentiation mechanisms and pathways within stem cells to choose from, our laboratory has the most experience with STAT3, which I will use as an example. As STAT3 knockout mice are embryonic lethal prior to neural development, the use of the model described here would enable generation of new and relevant data concerning the function of STAT3 with respect to stem cell differentiation into ectodermal or neural lineages. A summary example of the types of experiments that are necessary is as follows. (1) Use flow cytometry to sort cells that express a dominant negative form of STAT3, STAT3*β* [13]. (2) Analyze the differences between normal EBs and EBs constitutively expressing a splice variant of STAT3 and STAT3*β*, gathering data identifying how the absence of normal STAT3 activity affects the global process of differentiation. (3) Enhance and check outcomes using ESCs, iPSCs, or ASCs genetically modified to express GFP upon differentiation into neuroectoderm. As an additional control, these GFP-expressing cells would be analyzed for proliferation markers, confirming tracking of peak neural differentiation and not just an expansion of a neural precursor cell population. 

 In summary, what is being proposed here is a protocol built upon testing and validating a comprehensive ESC/iPSC/ASC differentiation model that could be adapted by other scientists for desired cell types, as well as a modeling approach that others could adapt to optimize the stepwise nature of ESC/iPSC/ASC experimental designs. Further, this model could be used to explore the role of pathways (e.g., STAT3 in ESC differentiation). This is a multidisciplinary approach that once implemented would yield methodological and scientific results that could have far-reaching impact, as well as providing the tools needed to move our own and others, research programs forward.

## 3. Comparing Statistical Methodologies: An Overview

There are two main approaches to statistical analyses: the current frequentist approach and the Bayesian approach. Yang et al. [21] described the comparisons of these two approaches nicely. The more commonly used frequentist approach is based on the philosophy of disproving the null hypothesis, in other words, showing no association between groups. Results of the frequentist analysis are summarized by a *P* value and confidence interval. Based on the *P* value, an effect is given to one of two classifications as either “significant” or “not significant.” Of course sample size can provide a buffer to the *P* value especially when there are outliers that heavily stray from the mean. On the other hand, when there are no outliers, a clinically trivial effect can be statistically significant when the sample size is large. As a result, *P* value can sometimes mislead readers and researchers [22–24]. It is up to the reader to scrutinize the data and critically evaluate the interpretation of the results that have been put forth either before the papre is published or when reading one that has been published.

In the second, the Bayesian approach, simply assigning whether there is arbitrary classification of significance or non significance through a *P* value, is not the outcome that defines whether the data can be used to affect change clinically or empirically. Instead, the Bayesian analysis can evaluate and assess the *certainty* (or uncertainty) of all or each data point within the data set being generated. A direct inferential statement on the uncertainty of the data or the interpretation of what has been analyzed is made after relevant data have been acquired and their contents interpreted and agreed upon. As a result, it is possible to use the Bayesian analysis to make clear decisions about the experiment almost in real time. For instance, if a risk reduction of 10% or more is considered clinically significant, the Bayesian analysis allows a statement to be made such as “there is a 90% chance that the relative risk reduction is clinically significant” [21]. This statement, also referred to as “posterior probability,” is considered more scientifically useful [24] than a *P* value, because it directly addresses the clinically relevant question. Moreover, the Bayesian method approach to scientific evidence can be considered in some respects a real-time statistical analysis because it is a constant process of learning and updating information, which allows the incorporation of prior data into the present data to arrive at a better conclusion. An effect or an association is continuously updated when new data become available [25], which can be considered equivalent to a meta-analysis. 

So, why switch from a frequentist approach to a Bayesian? Put as succinctly as possible, the Bayesian method offers a logical and direct inference on an effect [26], and perhaps equally as important, it is also useful in cases where repeated data collection is difficult or too expensive. 

The main idea driving the concept of applying Bayesian networks to better understand stem cell differentiation depends upon a simple notion of performing a minimal required set of experiments, followed by data acquisition and interpretation to understand how, when, and which signaling mechanisms are active resulting in the highest quality and the highest number of desired cell/tissue types. Yes, experiments are done first to acquire data, but once the data are analyzed by Bayesian statistics, a researcher then does not have to *always* run experiments to determine if that specific day is the best day to harvest cells for therapies. The Bayesian statistics enables the researcher to simply predict the best day to harvest cells from a batch of differentiating ESCs. Only from time to time do small samples need to be removed and analyzed to verify quality control. This latter point is key because it means that precious cells are not constantly in need to test the product as would be needed when using the more common frequentist statistical approach. Bayesian statistics helps to remove trial and error, which in stem cell research costs a lot of money and wasted time.

## 4. How the Bayesian Mindset Works with Different Biological Processes

### 4.1. Prediction of Chromatin Interactions

One of the most intricate set of molecular interactions within a cell is the protein-DNA binding complex. Genome-wide transcription as well as repression of gene activity is regulated by tens of thousands, if not more, of these dynamic DNA-binding events. The interaction between proteins and DNA can be tiered into three categories, DNA packaging by histones, sequence motif-specific direct binding (e.g., transcription factors), and indirect regulation by proteins such as scaffolding proteins or kinases [9, 27–30]. 

 Still poorly understood is the magnitude of interactions that regulates the multitude of chromatin structures that lead to gene activity, specificity, and silencing. One technique that is widely used to better understand these interactions is the genomic binding map. Existing maps have been generated in a number of different ways each of which serves distinctive purposes. For example, maps that show different proteins targeting a well-defined set of similar genes or the same gene can be very useful for identifying the mechanism(s) of binding. This information can then be used to develop drugs that prevent or promote binding for drug therapies. How are these maps made? One way is to show the binding empirically using such techniques as DNA fingerprinting or footprinting, while another may be chromosome immunoprecipitation (i.e., CHiP). Granted these techniques are highly accurate and well-accepted [31, 32]; however, they also are time-consuming, only identify a small number of interactions per experiment, and can be expensive for smaller laboratories. A good alternative is the *in silico* approach to identify chromatin components using a predictive search mechanism.

van Steensel et al. [9] devised a very nice Bayesian approach that defined a targeting interaction between two components as *X* and *Y* such that the presence of *X* at a specific set of genomic loci promotes the association of *Y* with those loci. They noted that this definition was functional rather than simply biochemical. The approach covered all three tiers of interactions including indirect protein protein-DNA interactions, which, to date, makes this analysis very powerful.

**Figure 1:**
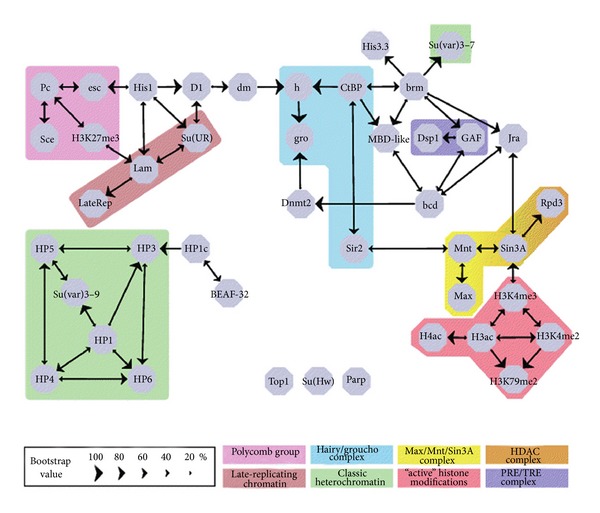
Bayesian Network model BN_80_ of the targeting interactions between 43 chromatin components. Nodes represent chromatin components; edges represent predicted targeting interactions with a bootstrap score (combined for both directions) of at least 80%. The size of each arrowhead is proportional to the bootstrap score of the targeting interaction in the corresponding direction. (from [9]) With permission from Cold Spring Harbor Laboratory Press.

 Using the drosophila genome and 43 chromatin components along 300 genes across chromosome 2, binding levels were analyzed by CHiP and scored by log2 ratios. They then employed the Bayesian network inference (BNI; [33–36]), which enabled the generation of a detailed interaction of all 43 components. The BNI in this case had two distinctive advantages for data analysis; (i) indirect correlations could be “explained,” for which they provided a straightforward example. If a protein A independently recruits two other proteins B and C to a common locus, those two proteins would map out as correlating with A *and* with each other. B and C are considered conditionally independent of each other with respect to A. (ii) The BNI was also able to predict the direction of the interaction. In other words did *X* target *Y* or *vice versa*? They termed this the causality direction [9].  Figure 1 is the result of this Bayesian approach for the 43 chromatin components. The authors went on to validate empirically some of the targets such as histones. Five BNI-predicted targets from Figure 1 were verified. 

 So, what exactly did this novel approach do for advancing the science of chromatin structure and regulation? Quite a bit (1) it provided a highly reliable and accurate tool for predicting many interactions that previously could have been missed or not found for years or decades; (2) it uncovered novel competitive targeting mechanisms and identified distinct chromatin remodeling enzymes necessary for opening up sites for DNA binding factors. In essence, this approach made the complex nature of chromatin interactions easier to understand.

### 4.2. Selection of Human Embryos for *In Vitro* Fertilization

IVF has become an important clinical alternative for couples who cannot, for some reason, conceive children normally. However, a reaction to problematic outcomes of IVF such as no births or the opposite, multiple births (e.g., octomom), as well as ethical challenges, governments have begun to regulate IVF clinics and procedures [37]. Consequently, embryologists have begun to search for approaches that would predict which embryos within the set obtained from the donor are the best to place back into the potential mother. Current procedures for evaluating embryos differ from country to country, but for most, a reliance of the clinic's resident embryologist to use a trained eye is most prevalent. This approach, as one would expect, is highly subjective, which tends to result in low or no birth rates. As one would guess, there are numerous variables in picking the best embryos morphology of the embryos is only one component of the choice. Age of the donor, sperm quality, egg dimorphisms, rate at which fertilization occurred, cleavage rate, number of previous cycles performed on donor, health of recipient, and many other factors go into choosing the best embryos for IVF. Ironically, artificial intelligence has begun to be used for increasing the success rate for artificial insemination [38, 39]. The first attempt at using less subjective methodology for embryo selection was a data mining technique employed on decision trees based on relationships between morphological features that had previously led to successful pregnancies and births [38, 40]. These first generation statistical/artificial intelligent protocols led to slightly more sophisticated algorithms based on pattern recognition and comparisons based on both human and computer predictions [41, 42]. However, a seminal paper by [37] proposed the use of Bayesian support system for embryo classification using multiple correlates such as multiple morphological aspects and clinical data of both donor and recipient (if different patients). 

 For IVF, acquiring Bayesian statistics begins with images of preimplantation embryos, which are then categorized by ~21 values including age of donor, number of previous cycles, zygote score (score type 1; all cells are equal size with no fragmentation of cells, to score type 5 100% fragmentation), thickness of zona pellucid, multinuclear or not, and many other examples [37]. These values can be numerical or yes/no and can be placed into software, now found free on the internet, that calculates the accuracy ± of a standard deviation for the desired outcome. In the case of IVF, embryos implanted can be measured to an accuracy of 0.9994 + 0.0589 on a scale of 0-1 [39]. The calculations to help prove validity of the Bayesian approach rely on receiver operating characteristics (ROC curves), which measure the cost-benefit ratio of diagnostic decision making ([39] and references therein). The ROC curve used for identifying embryos for IVF relies on the plot of sensitivity or proportion of embryo images correctly classified as being suitable for implantation versus specificity or the proportion of embryos that were misclassified as not suitable [39]. After surveying an *N* number of embryo image sets (*N* = 10 in [39]), a threshold is obtained. Finally a meaningful data point is obtained when the full area under the ROC curve is calculated (the AUC), usually by the trapezoid rule nonparametric method. What this means is that the Bayesian approach is an excellent predictor of choosing the correct embryo for transplanting into a foster mother. However, while the Bayesian approach looks promising for IVF, one caveat remains: publication of actual results. Both [37, 39] provide overwhelming evidence that the Bayesian formulas could be or perhaps should be applied to IVF clinical practices, to date, though no direct application of the method has been published. There are potential reasons for the lack of peer-reviewed data in the literature. One reason may be that image acquisition for each embryo is perceived as cumbersome; another is because data entry may be perceived as too time-consuming. I like to think the reasons lie in hesitation of trying a different method for data analysis and that applying the Bayesian methodology to IVF protocols is a relatively new idea in the literature. It simply needs time to settle into the IVF community. Time will tell.

### 4.3. Predicting the Toxicological Activity of Compounds

 The need is ever increasing for an accurate and efficient approach for predicting how drugs, toxins, and chemicals affect the environment or more specifically humans. Those driving the need for this predictive tool are three basic challenges that keep growing as human needs grow for food, clean water, and drugs to cure diseases. Those challenges include (1) a need to decrease the time it takes for hands-on experimental assays, (2) the ever-increasing costs, and (3) a need to decrease or replace animal testing [62, 63].

 Until about 2009, Descriptor-based quantitative structure-activity relationships (QSAR) models [64] were the predictor model of choice for toxicology [65]. However, the quality of those models depended on the quality of data used to develop the model and the limitation of the nature of the compounds used to develop the model [66]. The problem with the QSAR approach was that the model really only worked well on small sets of structurally related compounds and a single defined target. As a result, a better approach was necessary to overcome sometimes substandard predictive values and improve modeling of noncongeneric series of compounds. Huang et al. [63] assessed three methods novel to the toxicity field, weighted feature significance (WFS), sequential minimal optimization (SMO), and naïve Bayesian methodology. In short, while [63] were partial to WFS for prediction of toxicity, the Bayesian method was equally comparable in many respects. Perhaps other subtypes of the Bayesian approach (i.e., selective naïve Bayes, tree augmented naïve Bayes, or *k*-dependent Bayesian classifiers; defined nicely in [39]) may have been stronger predictors than WFS; however the point is made by [63] that the Bayesian approach is a valid one for predicting toxicological interactions.

## 5. A New, More Clearly Defined Model Would Benefit Prediction Outcomes of Specific Cell Types Differentiated from ESCs, iPSCs, and ASCs

The idea of stem cell based therapies is particularly prevalent in the fields of diabetes, cardiac and neurological disorders. These diseases are particularly good targets for stem cell therapy because the majority of the symptoms are associated with the loss of one specific cell type, for example, the dopamine (DA) neuron for Parkinson's disease (PD) or *β*-islet cells for diabetes. For PD, researchers in the past have shown that transplantation of fetal midbrain cells can temporarily alleviate PD symptoms in the rat model system [67, 68]. However, this effect is fleeting, as these cells do not *reliably* differentiate into DA neurons. Thus, researchers have turned to ESCs and iPCs, which can consistently be induced to form DA neurons *in vitro* [5, 8, 69]. 

**Figure 2:**
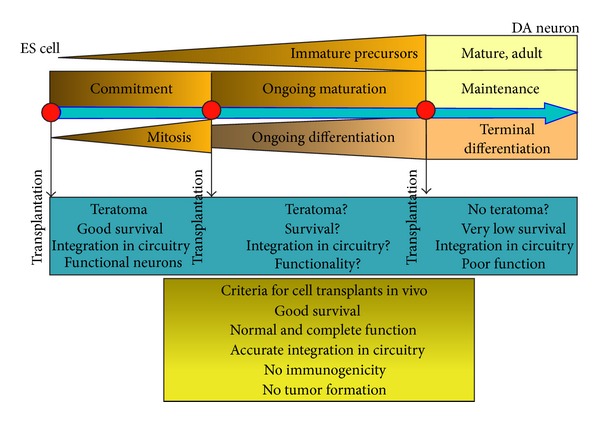
ES cell-derived DA neurogenesis and its implication for transplantation. *In vitro* and *in vivo* differentiated ES cells differentiate into immature neural precursors, which develop into mature adult DA neurons. During cell development, three major phases take place: commitment into the neuroectodermal cell lineage, maturation into the DA-specific neuronal phenotype, and maintenance of cell function. In these phases, the developed cells are either mitotic, for example, ES and lineage-committed immature precursors or terminally differentiated immature or mature DA neurons. For transplantation purposes, it is critical to determine the optimal state of the transplanted cells. For example, it has been shown that transplantation of ES cells in the brain showed good survival and differentiation into functional DA neurons with integration in the brain circuitry but partially developed into teratomas. Some of these observations have also been made by transplantation of immature precursors (unpublished observations). In contrast, transplantation of fully differentiated mature DA neurons did not reveal teratoma formation, but, poor survival and suboptimal function in the brain. These results demonstrate that certain criteria for a DA graft need to be fulfilled in order to achieve optimal results in future ES cell-based transplantation paradigms for PD, which can be summarized as follows: good survival, proper function, and integration into the brain circuitry with no immunogenicity and tumor formation (from [10]).

While the potential use of ESC/iPSC/ASC-derived terminally differentiated cells types as therapies is promising, there are several problems that are currently being addressed. These problems include formation of teratomas, grafting efficiency, differentiation capacity, incorporation into existing tissue (i.e., synapse formation for neurons or grafting potential of cardiomyocytes), and immunological response. These factors were summarized in an illustration from a relatively recent paper of stem cell therapies (Figure 2; [10]). This figure clearly depicts the need for a better understanding of neural specific ESC differentiation, including the identification of ectoderm-inducing factors and possible transitional cell types. 

In order to understand the progression of a specific differentiation outcome such as neural differentiation in the ESC model system, we must first examine differentiation as a whole. Differentiating ESC/iPSCs generally are induced to form EBs, which recapitulate blastocyst development but lack the inherent organization of the embryo. As in the embryo, ESC differentiation is thought to be an inductive process, in which development of each germ layer influences the other germ layers. However, the differences in organization between the blastocyst and the EB may influence the timing and efficiency of differentiation inducing signals [1]. Thus, for example, an understanding of neural differentiation alone is not sufficient. Researchers should understand neural differentiation within the context of generalized differentiation of their specific type of stem cell. One of the roadblocks in mapping out ESC differentiation is the broad range of cell culture protocols that are used [5, 10, 70]. Some procedures allow for the formation of EBs, while other protocols skip this step and induce differentiation without EB formation. Because the ability to establish ESC lines is dependent on strain specific Oct4 regulation [71, 72], all established mouse ESC lines exhibit highly conserved self-renewal and differentiation capacity. Therefore, when treated under the same conditions, stem cells from different mouse strains usually behave similarly [73], but this may not be the case for other types of stem cells. Standardization of differentiation techniques represents the first step toward truly understanding stem cell differentiation. Establishing a “normal” progression of ESC differentiation can then serve as a baseline comparison for gauging the success of tissue specific differentiation procedures.

### 5.1. Can Differentiation Be Modeled Using Bayesian Analytical Approaches? 

A brief perusal of the literature reveals only a few studies that have used Bayesian statistics to amplify the outcome of acquiring desired cell types ([74, 75] to be discussed below). However, one aspect of stem cells, the concept of differentiation, has been evaluated in other cell types using Bayesian methodology. Levels of differentiation have been investigated quite successfully using Bayesian statistics when added to the list of procedures for evaluating metastasis and tumorigenesis [76]. Lung cancer and pulmonary nodules are both radiologic abnormalities that are often detected incidentally. While most nodules are benign, some can represent stage I lung cancers that must be identified, classified, and distinguished from their benign counterparts. Furthermore, this procedure should be as cost effective as possible. In these cases, morphology presides as the method of choice for determining benign from malignant modules [77]. Using conventional imaging techniques such as thin-section computed tomography (CT), nodule borders, size, contour, distortion of adjacent vessels, and internal nodule characteristics can all be used to begin making an assessment. Unfortunately, not all small nodules with smooth, well-defined margins are benign resulting in false negative outcomes using CT technology alone. What is needed for cost-effective determination of lung cancer assessment is the combination of growth rate analysis, CT technology, biopsy, positron emission tomography when available, and a Bayesian approach to comprehend the data from all of these techniques [76]. 

In the plethora of cases where data analyzing indeterminate solitary pulmonary nodules must be evaluated and accurately characterized, Bayesian analysis allows more precise determination of the probability of malignancy (pCa) [78, 79]. Here, Bayesian analysis uses likelihood ratios (LRs) for numerous radiologic findings and clinical features associated with solitary pulmonary nodules to estimate pCa [78, 80]. The LR for a given characteristic is derived as follows:

(1) $$\begin{array}{c} \text{LR}=\frac{\text{number}\;\text{of}\;\text{malignant}\;\text{nodules}\;\text{with}\;\text{feature}}{\text{number}\;\text{of}\;\text{benign}\;\text{nodules}\;\text{with}\;\text{feature}}. \end{array}$$

An LR of 1.0 indicates a 50% chance of malignancy. LRs less than 1.0 typically indicate benign lesions, whereas LRs greater than 1.0 typically indicate malignancy. LRs for selected radiologic features of nodules and patient characteristics are thoroughly described in [77, 80]. The odds of malignancy can be calculated as

(2) $$\begin{array}{c} {\text{Odds}}_{\text{pCa}}={\text{LR}}_{\text{prior}}\;\;{\text{LR}}_{\text{size}}\;\;{\text{LR}}_{\text{sh}}\;\;{\text{LR}}_{\text{edge}}\;\;{\text{LR}}_{\text{calcif}}, \end{array}$$

where LR_prior_ is the likelihood of malignancy in all nodules based on local prevalence of malignancy and LR_size_, LR_sh_ (LR_smoking history_), and so on are possible additional variables. pCa is calculated as

(3) $$\begin{array}{c} \frac{{\text{Odds}}_{\text{pCa}}}{\left(1+{\text{Odds}}_{\text{pCa}}\right)}. \end{array}$$

Bayesian analysis has been shown to be superior to evaluation by experienced radiologists in the stratification of benign and malignant nodules and can be useful in determining treatment options. pCa for any nodule can be calculated with Bayesian analysis on Dr J. Gurney's Internet Web site at http://www.chestx-ray.com/ [last accessed November 17th, 2012]. 

In Erasmus et al. [80], LRs were calculated from four clinical scenarios, which evaluated hypothetical male patients with a smoothly marginated 7-mm nodule in the right middle lobe. These calculations resulted in models enabling distinct decisions for the most cost-effective strategy for management of a solitary pulmonary nodule depending on the pCa for that nodule for each patient. Several studies from the mid-1980s suggested that the most cost-effective strategy is observation when pCa is low (<0.05), immediate surgical resection when pCa is high (≥0.60), and biopsy when pCa is between 0.05 and 0.60 [81–84]. The data presented in [80] suggest that the most cost-effective management strategy for the first two patients with pCa's of 0.01 and 0.05, respectively was observation, whereas the most cost-effective strategy for the third and fourth patients with pCa's of 0.07 and 0.5 was biopsy.

### 5.2. More Proof of Point

Perhaps one of the most elegant and thorough investigations into mathematical modeling of a complex biological process such as cancer came from Andrey Rzhetsky's laboratory [85], which showed that theories (in this case about the process of metastasis) sometimes fail because of poor selection by experts in the field of assumptions about existing data. Scientific uncertainty about biological models seems to be the neglected stepchild of biomedical modeling, and this uncertainty only partially transpires in research publications. They go on to discuss that this uncertainty predominantly resides in experts' minds and close conversations, which I can anecdotally attest to every time I partake in deep discussions at meetings. However, they note that in some fields, such as engineering, it is more common to acquire expert opinion and employ probabilistic models when limited information is available [85]. Even there, however, the focus is on ascertaining modal opinion and not estimating diversity and uncertainty. 

 So, can divergent theories among experts be captured and modeled? Divoli et al. [85] argue that not only can they be captured and modeled, but the model can also be analyzed using Bayesian statistics to generate a formal probabilistic hypothesis against which researchers can evaluate data. What was illuminating in their paper was that the results they obtained did not match their expectations. They expected occasional disagreements, but instead encountered a wide level of conceptual diversity. They further anticipated that minor matters would generate mild deviation, but agreement upon the majority of issues. On the contrary, they found widely divergent and distinct stories about their topic of metastasis. One example of a large divergence included whether or not secondary tumors could lead to “tertiary” metastasis. Their in-depth Bayesian analyses suggested that a few ideas were widely shared and that many more ideas were rare and held only by individual scientists or small groups. They further show that fidelity for scientific assumptions from a very large number of experts would be required to approximate the collective knowledge of all experts. However, common assumptions could be captured from the analysis of a small number of interviews. Nevertheless, many research areas in biomedicine approximate these conditions, including investigations of virtually every complex disorder resulting from a combination of multiple genetic and environmental factors like schizophrenia, coronary heart disease, or asthma [85].

### 5.3. What about Modeling Stem Cell Differentiation Using the Bayesian Framework and Approach?

To improve predictive measures of ESC/iPSC/ASC, I am suggesting an approach to understanding stem cell differentiation based on the Bayesian perspective of statistical analysis. Applying this basic principle of a Bayesian approach to stem cells is not much different than previously described for other biological processes. Prior data are used to generate direct probability statements [86]. Thus, in contrast to the frequentist approach to statistics, which would estimate during differentiation that the probability of seeing one's data based on the assumption that the null hypothesis is true Pr(*A* | *H*_0_ = true), the “*P* value”, Bayesian methods would estimate the probability that the null hypothesis is true given the results of one's previous experiment Pr(*H*_0_ = true | *A*). The latter statement of probability, of course, is based on data or events that actually occurred (*A*, the observed data), whereas a frequentist statement of probability would be based on an assumption that the null hypothesis is true (unobservable). In the context of stem cells, null hypotheses would consist of the probability that differentiation occurred and of the timeframe for peak differentiation into each cell fate. This circumstance is similar to the application of Bayesian methods to the ongoing monitoring of many types of clinical trials [87]. That is, in this type of research, what “*H*_0_ = true” represents is either that differentiation occurred or that differentiation has peaked.

Crucially, when generating probability statements using Bayesian methods, the probability of the null hypothesis being true may change as more data is considered. This iterative property of the Bayesian approach makes it an ideal system for analyzing highly variable scientific data, such as ESC differentiation. A critical feature of this approach is that once a Bayesian model has been estimated, its utility in “predicting” the data is testable and very reliable. This is referred to as “posterior predictive model checking” [88]; the probabilistic model that the analysis leads to is the functional form of the “posterior” probability distribution. This function (model) is used to simulate a new “data set.” These new data are consistent with the Bayesian model (having been generated by that model), such that the model “predicts” this simulated data. When this simulated data set is compared to the original data set, the fit of the Bayesian model to the observed data is checked (“model checking”). If the model-implied data are consistent with the actual data, this is evidence that the Bayesian model does indeed fit the observed data. No such explicit model checking is available when non-Bayesian methods are used; the non-Bayesian alternative is a randomization test approach [89] that is not specific to the statistical or probabilistic model that the investigators hypothesize. As noted by [90, page 594], “…the traditional investigative method in the biological sciences should be complemented by the mathematical modeling approach… which can help to direct experimental research, while the results of experiments help to refine the modeling. The ultimate goal in the clinical setting is to use mathematical models to help design therapeutic strategies.” I believe the Bayesian approach when applied to stem cell differentiation embodies this philosophy explicitly.

 Two elegant studies recently used Bayesian networks to identify pathways during ESC differentiation [74, 75]. The objective of the work by Woolf et al. [74] was to identify how mESCs responded to extracellular stimuli by comparing the expression/activity of certain signaling pathways during self-renewal and differentiation. Using four extracellular factors, leukemia inhibitory factor (LIF), fibronectin (FN), laminin (LAM), and fibroblast growth factor 4 (FGF4), they applied cell growth and differentiation data to a factorial screen. Oct4 was used as the differentiation reference marker, and single-cell seeding was used for measuring proliferation. Western blot analysis using the highly versatile KPSS1.1 screen from Kinexus Inc. (Vancouver, Canada), which we have also used [13, 19, 91], provided phosphorylation state data. Their analysis using more standard methods such as Western blot analyzing p705 specifically on STAT3 was a nice added control [74]. 

So what did they find? First, Woolf et al. [74], indeed, confirmed previously reported signaling activities related to self-renewal (e.g., LIF/STAT3); however, perhaps more importantly, they found novel signaling mechanisms such as the role for Raf phosphorylation in differentiation and proliferation. The strength underlying their work was their demonstration that linear, nonlinear, and multistate logic interactions connect extracellular cues to intracellular networks, which control self-renewal, proliferation, and differentiation. 

The math behind their [74] statistics is involved but straightforward, and because they explained their methodology clearly and concisely, I did not want to rewrite mathematical equations. Their models, however, did warrant reviewing. Their Bayesian-based models were strong because they included three points: validation, visualization, and the ability to predict “what if” experiments. Validation of their model was done by shuffling data sets and calculating *P*(Data l Model), which is known as the probability of a model given data [74]. The arithmetic equation to calculate *P*(Data l Model) consists of plugging data (e.g., number of Sox3 + cells) into specific variables of the equation. One of the important concepts of the equation is that each variable, also called a node, can be causally connected (represented by an arrow) to another node. Thus, each node has a list of parent nodes, which also can have a number of variables. With the equation in [74], specifically their fifth equation, each node could be measured with respect to their biological characteristics such as levels of protein phosphorylation or rate of cell differentiation. Of course they had to define the probability of a model, *P*(Model), so that it could have a numerical value, in this case, 0 or 1, signifying that the network of nodes was (assigned a score of 1) or was not (assigned a score of 0) allowed. Software does exist for generating the *P*(Model) and thus the *P*(Data l Model) upon request from Woolf et al. [74]. The data can then be visualized using Graphviz from http://www.graphviz.org/ (Last accessed November 17th, 2012). To this end, a large score difference between shuffled and unshuffled datasets indicate that a reliable prediction of signaling pathways can be made. 

Testing their Bayesian network model, Woolf et al. [74] compared data from known biochemical signatures to the Bayesian prediction from the initial KPSS1.1 screen and found strong correlation with the LIF/STAT3 pathway and undifferentiated cells. LIF activates STAT3 through its receptor gp130, which recruits and activates JAK2 via phosphorylation, which, in turn, phosphorylates STAT3 on tyrosine 705. This phosphorylation event causes STAT3 to dimerize, enter the nucleus, bind to DNA, and activate genes. While their Bayesian network model did, indeed, predict a strong interaction between LIF and STAT3, it also determined which signaling pathways ESCs utilized for proliferation and/or differentiation. 

Three states of differentiation were analyzed: (i) the undifferentiated cell proliferation rate, (ii) the differentiated cell proliferation rate, and (iii) the differentiation rate. What they found was that the rate of differentiated cell proliferation was governed by laminin and the phosphorylation states of RAF1 and p38*α*MAPK, while the undifferentiated cell proliferation rate was governed by LIF and p38*α*MAPK. They were surprised to see LIF as a predicted regulator of undifferentiated cell proliferation because it had been thought to be an anti-differentiation agent, and as such they suggested that LIF may activate another unknown or unmeasured pathway [74]. Their third criterion, the rate of differentiation, was predicted to be regulated by the phosphorylation state of Adducin*α* and Erk2. The biological implications of these components, especially Adducin*α*, to drive differentiation in ESCs are not fully known; however, they are good candidates to test in further detail at the bench. 

The work by [74] revealed for the first time that ESCs are an excellent model system for Bayesian networking, in this case identifying signaling networks and their influences on differentiation. Perhaps one of the more important revelations from using this networking model was the ability to visualize data as a directed graph allowing for concise interpretation of large, noisy, biological data sets. 

### 5.4. Using a Clinical Trial Approach to Monitoring Stem Cells

In addition to this general approach to analyzing ESC/iPSC differentiation model, since the entire framework of this approach is Bayesian, a clinical trial monitoring perspective is used to predict the probability of reaching the clinical trial goal (at the end of the trial) by sequentially analyzing the data as it is collected (monitoring). Although clinical trial monitoring is typically tailored towards identifying stopping rules (i.e., when the probability of harm or efficacy is great prior to the planned end of the study), this framework could easily be adapted for stem cell differentiation so that change can be monitored over the course of the research. As pointed out by [92, 93], using a clinical trial data monitoring approach from a Bayesian perspective permits the termination of the clinical trial (or the end of the experimentation), when the results of an analysis conducted prior to the completion of all data collection suggest a high probability (higher than some priori threshold values) that the outcome will be positive or negative, given the null hypothesis of the overall study (p. 2180). For stem cell research, this clinical trial monitoring approach is important, since without it stem cell research is simply a planned series of incremental experiments, *all* of which must be completed since a decision or “model” can only be created, currently, based on the completion of *all* planned experiments. The monitoring perspective implies greater efficiency because as the model evolves, the estimates also evolve, until sufficient information is obtained (to characterize the “state of knowledge”) about the ESC differentiation model and time course. This corresponds to “early stopping” of clinical trials, whether for futility or superiority of a new treatment—the decisions to stop early are based on a Bayesian analysis and on the monitored data. For example, at first, data from Western blots, RT-PCR, and immunofluorescence can be used to estimate the peak day for differentiation of ESCs. Then, using this estimate as the null hypothesis, more detailed flow cytometry experiment can be conducted. Results from the flow cytometry would allow more refined and updated estimates of the peak day for differentiation as experiments are replicated that could be retested by Western blot, for example, to verify the refinement. This process may continue through at least two iterations, using triplicate measurements, permitting focus to be placed on time course of the actual peak day for differentiation, while accounting for variability in differentiation. The replications are treated as interim analyses (monitoring), continuously update a probabilistic model. Lastly, this type of work usually requires a team effort optimizing both the cellular modeling expertise and the statistical expertise of the two PIs.

### 5.5. What about Using Bayesian Networking to Predict Reprogramming?

This was an eye-raising question asked by a colleague, recently. I thought this was a great question because a number of methods for generating iPSCs has both advanced the science of iPSC generation and clouded it at the same time [94–101]. Which method is best? *Because* three of the five or six methods for generating iPSCs were published quite recently [98–100], does this mean that more procedures have yet to be discovered? Answering these questions has not been trivial primarily because of the methodology currently used by most investigators to generate iPSCs. A pool (~20 or more) of reprogramming factors is usually identified (usually from the literature) and then tested *in toto* to determine if they are, indeed, capable of inducing a given cell type to become pluripotent. If successful, this procedure is then duplicated but with sequential removal or addition of specific groups or individual members of the original factors. I remember reading [94] and thinking how elegant the work was but also wondering how many times they failed and how much all the work involved in that paper must have cost. 

Answering the questions posed above for generating and procuring iPSCs when so many new protocols are coming online requires a different approach, one that is both systems based and computational for predicting the components within inducing “recipes.” In late 2011 and 2012, the Bayesian networking approach was employed, for the first time, and while application of this method for development of iPSCs is still very new [102–104], the initial reaction is that the criteria and the models work nicely. Chang et al. [102] used Bayesian networking to search for optimal reprogramming recipes that result in more efficient reprogramming and better quality iPSCs. Their model also allowed for monitoring the trajectory of reprogramming from a fully differentiated cell to the iPSC.

Their methods, while very involved, can now be used by anyone to predict recipe components for reprogramming. Their initial analyzed genes known to be involved in reprogramming (e.g., Oct4, Sox2, Klf4, cMyc) and genes unknown to be involved in reprogramming (e.g., Prdm14). They specifically employed dynamic Bayesian networking (DBN) unrolling the cyclic human ESC network first and then applying those network interactions to search for recipes. 

The order of events for generating recipes begins with an in-depth literature search to collect information regarding markers that drive pluripotency and markers the drive differentiation in hESCs. With this information, a genetic network can be constructed manually revealing the connections between genes that regulate pluripotency and genes that regulate differentiation. Chang et al. [102] focused on 52 different genes including Oct4, Nanog, and Sox2, the three key genes regulating pluripotency. Although Chang et al. [102] focused on genetic markers, as mentioned above, the fact that a number of other non-gene-oriented procedures for generating iPSCs now exist (e.g., specific miRNAs) means that initial networks could be generated using genes, miRNAs, chemicals, proteins, or any combination of these [94–101]. 

 Once the network (a gene network in [102]) is generated and agreed upon, the next event entails each gene being treated as a binary variable, that is, active or inactive. DBN is then applied in order to model activity of feedback loops in the network. This is important because while a cyclic network generated from the literature provides information about gene interactions, it is static. Cyclic networks do not allow for the stochastic nature of ESCs, but DBNs do. To generate the DBN from the cyclical network, a series of acyclic graphs are created based on whether a protein emits or receives information from another protein. Acyclic graphs of all genes in the network (52 genes for Chang et al. [102]) are then organized into a 2-time slice Bayesian network (2TBN), which can be updated with data when they become available  [102]. The next step is developing the parameters that are assigned values. Each parameter becomes an instance within the DBN model, which when averaged together allows inferences to be made. Inferences can then be calculated resulting in the probabilities of all gene/protein interactions in the agreed-upon network. This model was successful for identifying recipe components for reprogramming cells to pluripotency. Looking at 22 genes, [74] found two states that had significantly higher probabilities than any other state; the two states corresponded to the stem and the differentiated state, respectively. From these 22 genes, using the root mean square deviation (RMSD) and the Pearson and Spearman rank correlation coefficients comparing reprogrammed cells to hESCs, expression similarities and reprogramming efficiencies for at least 163,185 possible combinations of genes were calculated. Out of all the possibilities, 962 recipes resulted in efficiencies greater than 0 or, in other words, possible recipes for reprogramming. The most optimal possible recipes had both high efficiency and high expression of genes similar to hESCs (i.e., low RMSD and high Pearson and Spearman correlation coefficients). Their top candidate recipes were separated into three categories each comprised of master regulator genes plus two other genes that were predicted to increase programming efficiency. Oct4 was indispensable as it was found in all recipes. Sox2 and Nanog were also indispensible, but could replace each other, while genes like LMCD1, PRDM14, PBX1, KLF4, ZFPA1, ZNF206, FOX1A, GDF3, ZFP42, TDGF1, and ZIC3 varied with respect to which master regulators they were paired with. 

 In essence, this work and others have now shown that, by doing experiments a minimal number of times and comparing those findings to preexisting data in the literature, dynamic analyses using BDNs can predict the best combinations of genes, miRNAs, proteins, or a combination of all of these to obtain the best quality iPSCs in the most efficient manner. 

## 6. Delving Deeper into the Bayesian Model for Stem Cells: Markers and Definitions

Based on a detailed review of the current literature, seven markers are generally considered a strong minimal representation for the minimum needed to define the differentiation of ESC/iPSCs/ASCs into cells/tissues representing the three primary germ layers (Table 1). In addition to identifying the endoderm, ectoderm, and mesoderm, these markers can also characterize the transitional cell types of mesendoderm and neuroectoderm (Tables 2 and 3). Examples of markers and cell type definitions are detailed in the following tables (Tables 1–3).

**Figure 3:**
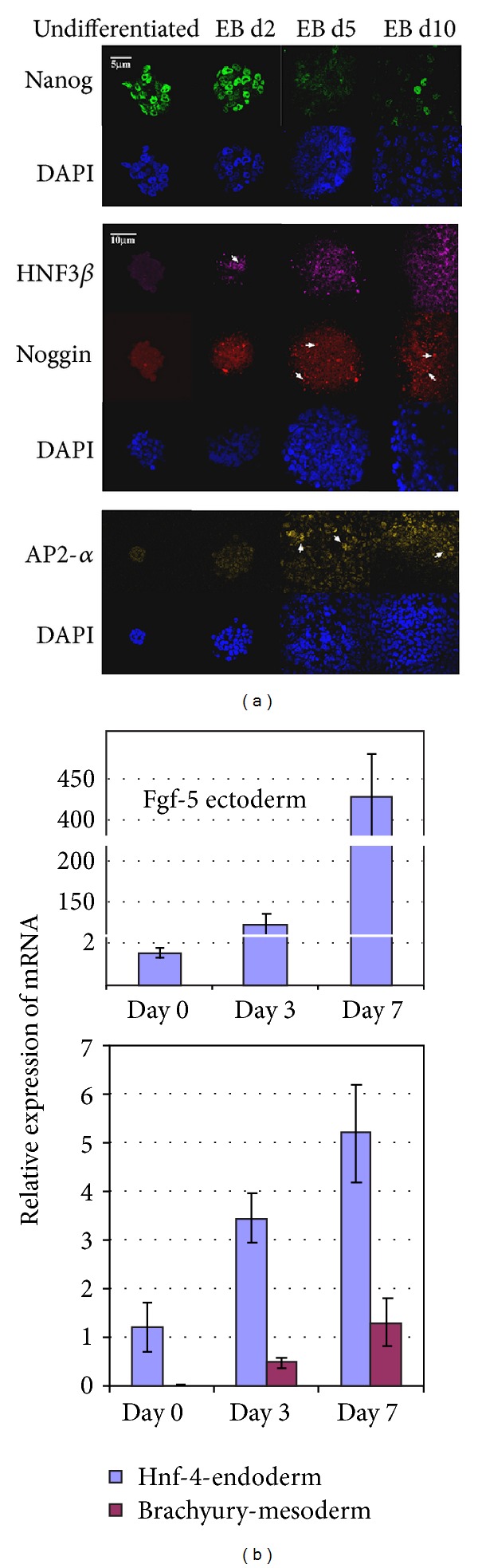
The data needed for Bayesian network equations can be easily acquired. Here, confocal analysis and qRT-PCR are examples of data acquisition that can be used to generate plots such as those found in Figures 4 and 6. (a) Immunofluorescence shows expression of Nanog in the nuclei of undifferentiated cells. This expression decreases drastically after 2 days of differentiation. HNF3*β* expression is first visible at d2 in suspension. White arrowhead points to nuclear localization of this transcription factor. By d10, expression is localized to the right half of the EB. Noggin expression is first seen at day 5 and, as expected of a secreted factor, localizes to vesicles in the cytoplasm (white arrows). At day 10 Noggin is confined to the left half of the EB. Ap2-*α* is first detected at d5 in suspension. Like the other markers, by d10 Ap2-*α* shows specific staining in one portion of the EB. 5 *μ*m scale applies to all images in upper panel. 10 *μ*m scale bar applies to all images in lower panel. (b) qRT-PCR of EBs at specified days of differentiation illustrates one method for understanding timing of germ layer development using germ layer-specific markers Fgf5 for ectoderm, Hnf-4 for endoderm, and Brachyury for mesoderm.

Immunofluorescence must be conducted to confirm the expression of each marker during differentiation. This process, which should use the same antibodies and basic staining protocol as flow cytometry, represents a control for the specificity and efficacy of each antibody and for the reliability of future experiments. Nanog expression is highest in undifferentiated ESCs, as expected, and decreases throughout differentiation (Figure 3(a)). Interestingly, some Nanog positive cells still remain at d10 and may represent those undifferentiated cells that contribute to teratoma formation. The endoderm marker Hnf3*β* is first expressed at approximately day 2, while the mesoderm and ectoderm markers, noggin and Ap2-*α*, respectively, are not present until d5 (Figure 3(a)). Strengthening confocal data, our quantitative RT-PCR results clearly show the general time course of expression for specific markers (Figure 3(b)). Taken together (Figures 3(a) and 3(b)), these data suggest that differentiation of each germ layer proceeds over a varying time course, and changes in staining intensity over time leads us to believe that peaks in differentiation can be determined. 

Combined, the immunofluorescence, Western blots, and RT-PCR confirm that the cells do, in fact, differentiate into each primary germ layer, based on the presence of each marker. Although these data cannot be used to generate a quantitative measure of the number of cells differentiating each day into each tissue type, this approach does enable the generation of probability statements and descriptive statistics that address the variability of both ESCs/iPSCs/ASCs and their application to Bayesian methods of analyses.

## 7. Delving Even Deeper: Steps for Completing the Bayesian Model

 The experimental design is based on an iterative Bayesian framework (IBF), in which preliminary data can be used to generate a predicted outcome for a future experiment [86]. This process, explained in Table 4, is ideal for narrowing the focus of a desired time course, increasing the resolution of the analysis, accounting for variability in our system, and maximizing data output while minimizing costs. Each time the experiment is conducted, a new outcome, which is more reflective of the data, will be calculated. Based on this new prediction, the experimental design may be altered to home in on the peak time-point for fate specific ES cell differentiation.

**Figure 4:**
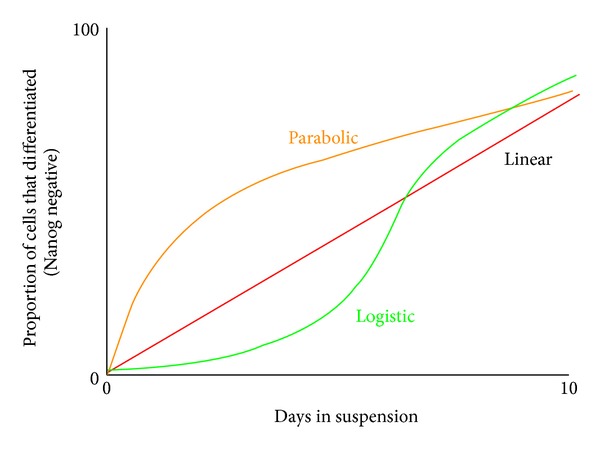
This theoretical curve fitting graph reveals the type of data that would come from plotting the number of cells that have differentiated over time. Data like those found in Figure 3 could be used to generate a graph such as this. Because differentiation is regulated by inductive signals, we predict it is modeled by a logistic curve; the lag period caused by upregulation of signals, the growth period caused by high signaling activity, and plateau caused by downregulation of signals.

*Step 1: Undifferentiated versus Differentiated Flow Cytometry.* Each individual cell must first be categorized as “differentiated” or “undifferentiated” based on the definitions stated above (Table 2). These data (cell counts) are then graphed over time, and the proportion of cells that differentiate on any given day is calculated. These data are subjected to curve fitting analysis using a software program such as GraphPad. While this does not give us insight into the time course of tissue specific differentiation, it does allow graphical modeling of the process of differentiation and determining the proportion of cells that actually differentiate on any given day (Figure 4). Based on the shape of this curve, this graph would identify time points at which differentiation is the most probable. These time points may later be used by researchers to enhance stem cell culture differentiation protocols. Eventually, this curve, combined with the data from step 2 (below), creates a cohesive and comprehensive model of the time course of stem cell differentiation. From a Bayesian perspective, the research begins with a “noninformative prior” using an average over previously published results, as the starting estimate for when a peak might be expected in differentiation. As data are collected, this estimate is updated; as the graphical model (Figure 4) emerges, the functional form of the probability distribution also emerges. This distributional form is combined with the increasingly precise estimate of the peak in order to achieve both a model that can be used to achieve posterior predictive model checking [88], which would validate the resulting model, as well as obtain credibility intervals for an estimated peak and the time course milestones. Beginning with a “noninformative prior” would yield credibility intervals that have a similar interpretation as confidence intervals would under a frequentist approach [93]. 

One major concern in the generation of a model is its applicability. To test that this model could be used to describe the differentiation of different ESC types, we followed feeder-free CCE and feeder-dependent R1 ESCs as they were induced to differentiate [13–15]. Using an easily detectable output, the differentiation of functional beating cardiomyocytes, we determined that both cell lines differentiated along the same course of time [20, 91]. Other laboratories have also shown similar results when tracking ESC cardiomyocyte differentiation using molecular markers (references within [91]). Together, these data suggest that this model could be useful for researchers working to streamline differentiation of various cell lines.

**Figure 5:**
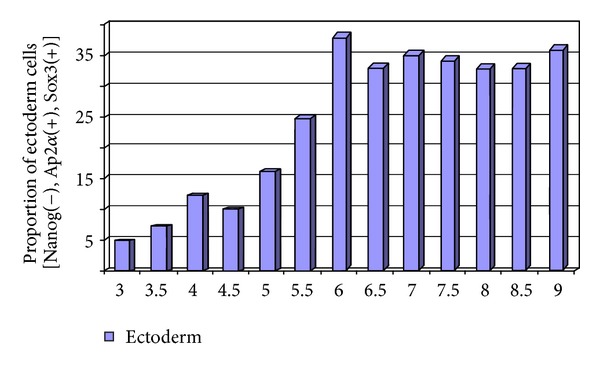
Possible outcome for ectodermal differentiation. In this example, using the differentiation markers AP2a and Sox3, the predicted peak for differentiation would be day 6, with a standard deviation of 3 days. The analysis should be done in 12-hour intervals.

*Step 2: Cell Type Specific Differentiation.* Taking the FACS/Baysian approach a step further, a window for obtaining the most pure population of a desired cell type emerges. Each cell is analyzed for the presence of each germ layer marker on the predicted mean day, plus or minus each day included in the standard deviation (Figure 5). For example, if the predicted mean day for differentiation is day 5 and the standard deviation is 2 days, then cells should be analyzed on day 3, 4, 5, 6, 7, and 8. For each day in this range, three replicates are analyzed, and the data points generated are used to calculate the mean and standard deviation for the number of cells that differentiated into each germ layer. The day at which the number of differentiated cells is maximal and the standard deviation is minimal then replaces the original predicted mean for the next iteration of data collection. For the second iteration of this experiment, the span of time over which differentiation is analyzed should be narrowed to increments of 12 hours. This increment reduction is appropriate because the estimation of the mean and standard deviation now becomes more accurate, as determined by the first round of data collection. The mean and standard deviation generated in this iteration becomes the final prediction for the day at which differentiation into each germ layer is most probable. Because this is the only value that will be considered at the end of the experiment, it is important to have enough replicates to generate descriptive statistics and to construct reliable credibility intervals. Five replicates are suggested. While further iterations may be able to pinpoint the highest probability of differentiation down to the hour, these data are not necessary because some cells within an undifferentiated culture will always begin to differentiate. As a result, this system contains too much inherent variability to be resolved over small increments of time. In addition, researchers, whether in the laboratory or the clinical setting, do not work 24 hours a day. Therefore, defining the process of differentiation within the scope of an appropriate work schedule will be more useful and powerful.

## 8. Applying the Model: Determine the Specific Role of STAT3 in Stem Cell Differentiation and Determination of the Ectodermal Fate

The experiments described above are meant for creating a much needed tool that researchers can use as a control for their research answering the question: when is the optimal day to sort my differentiated stem cells to obtain the most of my desired type of cell (e.g., cardiomyocytes, neurons, *β*cells, etc.). So, how could this be applied to researching a pathway from a purified set of differentiated cells? In Woolf et al. [74], the effects of external stimuli on intracellular pathways that drive differentiation were analyzed. Here, we look at more specific routes of differentiation primarily because the exact course of ESC, iPSC, and ASC differentiation is not fully understood, and the effects of certain proteins or drugs on the progression of differentiation are difficult to measure accurately. Thus, applying the Bayesian model to stem cells differentiation (here ESC differentiation) would establish a baseline or control time course for differentiation to a directed cell type. One of our goals over the past decade has been to identify the role of STAT3 in fate specific ESC differentiation and to demonstrate that our model can be easily and effectively manipulated. More specifically, a plan has been to focus on the effects of STAT3 on ectodermal differentiation. As STAT3 is known to be important for neural precursor formation, changes to this signaling pathway may be valid targets for increasing the production of ectodermal cell types and neural precursor cells for stem cell therapy [14, 105, 106].

### 8.1. Understanding the Role of STAT3 in ESC Differentiation

To better understand STAT3 function during differentiation, we are currently applying data from cell lines that exhibit drastically reduced STAT3 activity to the Bayesian model and comparing those data to control the set of cells. Data is gathered using FACS. These stably transfected cell lines overexpress a construct for STAT3*β*, a dominant negative form of STAT3 [107, 108]. The STAT3*β* cells lines express normal levels of the stem cell markers, Oct4 and Nanog, but markedly lower levels of the STAT3 target genes Cyclin D1 and myc (unpublished data and [13]). As our Western blot data has demonstrated, the efficacy of this dominant-negative experiment with this cell line allows us to compare the progression of differentiation in STAT3*β* cells to that of normal ESCs, thereby elucidating the effects of the loss of STAT3 of ectodermal fate commitment. Using a repeated measure ANOVA, it can be determined if the expression of STAT3*β* causes a statistically significant change in differentiation of any of the germ layers over time. 

To confirm the above data and to enhance our ability to clearly analyze the efficiency of ESC differentiation into ectodermal/neuroectodermal cells, for example, genetic perturbation techniques can be employed to introduce a transgene expressing GFP under the control of the Sox3 promoter to both wild type and STAT3*β* ES cells. These cell lines allow flow cytometry along with survival analysis to identify the exact time point at which commitment to the neuroectodermal fate occurs. Generally speaking, survival analysis is an advanced statistical approach for determining the competing risk of an event of interest occurring over time [109]. In our case, the event of interest is differentiation into the neuroectodermal fate, and the timeframe is the course of differentiation. 

**Figure 6:**
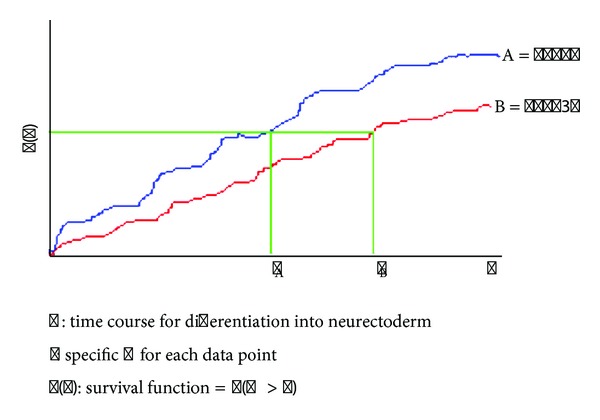
Theoretical sample of survival analysis data comparing the time it takes for wt CCE-GFP and STAT3*β*-GFP ESCs to commit to the neuroectodermal fate (i.e., time Sox3* promoter*-GFP is first expressed). Once differentiation data is acquired, the data could be plotted using graphviz at http://www.graphviz.org/ (last accessed 11/1/12).

 An added benefit of survival analysis is that, in addition to identification of neuroectodermal differentiation, one can also account for cell death (by PI staining) and differentiation into a nonneural cell type. Thus, the use of survival analysis in analyzing our GFP ESC lines should generate a graphical display of the time it takes for cells to commit to the neuroectodermal fate, providing a relative measure of the differentiation efficiency. An example of possible survival analysis output is depicted in Figure 6.

**Figure 7:**
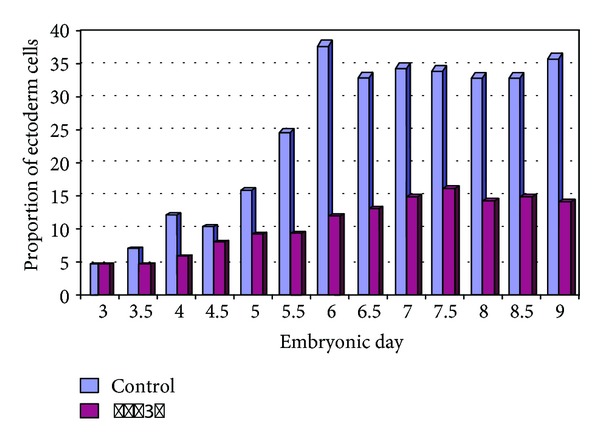
Possible outcome for control versus STAT3*β* comparison. We predict that STAT3*β* will reduce ectodermal differentiation.

Once the time course of STAT3*β* ESC differentiation has been established using the Bayesian model and compared to the control ESC differentiation Bayesian model, the data can be used to develop new techniques for differentiating ESCs down cell type specific pathways. For example, the data may show that the loss of STAT3 during differentiation negatively affects the production of ectodermal cells (Figure 7). Using this knowledge, additional flow cytometry experiments, in which STAT3 activity is upregulated at different points during differentiation, could be conducted. Again, by comparing these results to the control model, the effects of altered STAT3 activity on the efficiency of ectodermal differentiation could be determined and analyzed for statistical significance. In this way, other researchers could use our model to generate the most efficient tissue culture protocols for inducing differentiation of stem cells into any cell type of interest. The use of the Bayesian methodology will prove to be valuable for those interested in stem cell therapy, as it will eliminate the need for repeated cell sorting during differentiation, and it will allow cells to differentiate in a well-characterized, efficient and biologically sound manner.

## 9. Discussion of the Model: Breaking Down the Basics

 “Bayesian methods support sequential learning, allow for finding predictive distributions of future results, and enable borrowing strength across studies.” [86, 92] articulated the derivation of predictive probabilities that are computed in clinical trial monitoring from the Bayesian perspective when investigators are interested in stopping a trial (due to futility (no difference between two groups), or superiority (one treatment is better than the other)). The derivation and formulae for the case of a binomial variable are presented on pages 2180–2184 of [92] (with additional derivations in their appendix (page 2194)). Rather than recapitulating these derivations, I focus on the assumptions and details of the Bayesian approach in terms of how estimates are updated, and how it is known when data begin to converge on a specific estimate for differentiation and its time course. 

 Specifically, as stated above, a Bayesian analysis consists of three main components: the prior distribution, the likelihood of the data, and the posterior distribution [110]. The posterior is proportional to the product of the likelihood and the prior distribution. The posterior distribution is the joint distribution of the parameters being estimated; in the present context, given a binomial outcome (differentiated/did not differentiate), we would assume a normal approximation to the binomial distribution, and then the modes of the marginal posterior distributions will yield point estimates of the parameters (analogous to the sample mean and variance). Choosing the prior distribution, then, can be the stumbling block for either beginning a Bayesian analysis or packaging it for review (see, e.g., [93]). As recommended by [92], and given the binary (peaked, did not peak; differentiated, did not differentiate) nature of the outcomes to model, the beta distribution is chosen as the prior to make this as close to a “null” distribution as possible. Initial parameters should be chosen for the beta to both be 1.0. Thus, the prior distribution follows *B*(*α*, *β*), and before data are collected, it is assumed that *α* = *β* = 1. If the result of one experiment is called *S* (result of current experiment) and the result of the next experiment is called *T*, then Dmitrienko and Wang's equation enables one to estimate the probability associated with the result of an as-yet-undone experiment given the result of the current experiment (page 2183):

(4) p~(ts)=P(T=tS=s)=B(s+t+α,N−s−t+β)(N−n−t)B(t+1,N−n−t)B(s+α,n−s−β),

where $\tilde{p}(t|s)$ represents the predicted probability of *t* (the result of the next experiment) given *s* (the results of the current experiment), *N* = the total number of cells that can differentiate, *n* = the number of cells not yet differentiated, and *t* must fall between 0 and (*N* − *n*). Then the posterior event rate (p*) will be conditioned on both *S* = *s* and *T* = *t* and will also follow a beta distribution, but the parameters α* and β* will be found as α* = *s* + *t* + *α* and β* = *N* − *s* − *t* + *β*. This permits updating over all *T* and *S*. I have tried to simplify all of this work in Table 4.

### 9.1. Possible Problems and Solutions

Because the overall framework of the research proposed here is Bayesian, it is proposed to update estimations (regarding differentiation and time course) according to a Bayesian clinical trial monitoring method outlined by [92]. However, two problems can arise. Firstly, the evidence from different markers within each experiment or step might not be consistent. That is, some markers might support one conclusion, while other markers support another. This is not a problem for the science itself, but would require adjustments to the analytic plan. Namely, one would need to construct models to accommodate the diverse outcomes. This could be done in WinBUGS by establishing multiple chains with different starting values that represent the divergent outcomes (if they are observed). A second problem would be if assumptions about the prior distribution exert undue influence (that cannot be overcome by the data). To detect if this occurs, a sensitivity analysis to estimate the influence of a set of prior parameters should be carried out. This involves a variety of starting values for *α* and *β*, using an actual uniform prior distribution (instead of *B*(1,1), which would just *look* uniform) and tracking the results from each experiment to determine if the outcomes are consistent with what the literature suggest. For example, vastly divergent results on successive experiments would be identified, such that we would not simply continue to update without considering the reasonableness of each value of *S*.

## 10. Concluding Remarks

 Some of the data that I have written about here has not been previously published. I believe that this approach should be shared among the scientific community because if implemented, the IBF methodology could significantly improve both the collection of and understanding of cells differentiated from ESC/iPSC. I am not saying that the frequentist approach for analyzing stem cell differentiation should be abandoned. On the contrary, it has resulted in expert agreement in a number of concepts such as genes necessary for pluripotency [111–114]. However, there is still much for the experts to be learned from ESCs, iPSCs, and ASCs as they differentiate into desired cell types, and Bayesian statistics will undoubtedly assist the experts acquire the best cells of interest to, hopefully, speed up the process.

## Figures and Tables

**Table 1:** Tissue specific markers.

Cell/tissue type	Marker	Reference
ES cell	Nanog	[43] Mitsui et al., 2003; [44] Chambers et al., 2003
Endoderm	Hex	[45] Bort et al., 2004; [46] Chapman et al., 2002; [47] Heo et al., 2005; [48] Baron et al., 2005
Mesendoderm	Foxa2	[46] Chapman et al., 2002; [48] Baron, 2005; [49] Tada et al., 2005; [50] Kubo et al., 2004
Mesoderm	Tbx6	[47] Heo et al., 2005; [51] Chapman et al., 2003; [52] Uchiyama et al., 2001; [53] White et al., 2005
Mesendoderm	Noggin	[46] Chapman et al., 2002; [54] Sela-Donenfeld and Kalcheim, 2002
Ectoderm	Ap-2*α*	[55] Luo et al., 2002; [56] Mitchell et al., 1991; [57] Philipp et al., 1994; [58] Puelles et al., 2005; [59] Wood and Episkopou, 1999
Neurectoderm	Sox3	[47] Heo et al., 2005; [58] Puelles et al., 2005; [59] Wood and Episkopou, 1999 [60] Brunelli et al., 2003; [61] Pfeffer et al., 1997

**Table 2:** Undifferentiated versus differentiated.

Outcome	Nanog	Foxa2, Noggin, Ap-2*α* (antibody cocktail)
Undifferentiated	+	−
	+	+
Differentiated	−	−
	−	+

**Table 3:** Cell fate definitions (individually conjugated antibodies used together).

Outcome	Nanog	Foxa2	Hex	Noggin	Tbx6	Ap-2*α*	Sox3
Undifferentiated	+	±	±	±	±	±	±
Mesendoderm	−	+	−	+	−	−	−
	−	+	−	−	−	−	−
	−	−	−	+	−	−	−
Endoderm	−	+	+	−	−	−	−
	−	−	+	−	−	−	−
Mesoderm	−	−	−	+	+	−	−
	−	−	−	−	+	−	−
Ectoderm	−	−	−	−	−	+	+
Neuroectoderm	−	−	−	−	−	−	+

**Table 4:** 

Preliminary step: Western Blots, RT-PCR, IF		Research goals and progress
Markers	All (Tables 1 and 3)	(1) Confirm that all markers are expressed and differentiation of all cell types is occurring; replace any antibodies that do not work; revise differentiation protocol if necessary
Time course	Each day, d0–d10	(2) Determine the peak day for expression of each marker
Hypothesis	Nanog decreases by d2. Endoderm peaks at d3-d4. Mesoderm and ectoderm peak between d6-d7	(3) Determine a day to be used as + and – control for flow cytometry
Possible problems and solutions	Markers are expressed in undifferentiated cells— they cannot use ES cells as negative control	(4) Determine whether markers for each cell type are expressed at similar times or have varying temporal expression

Step 1: Flow cytometry 1		Research goals

Markers	Differentiated versus undifferentiated (Table 2)	(1) Using + and – controls from preliminary step, confirm that all antibodies work for flow cytometry; replace any antibodies that do not work
Time course	Each day, d0–d10,	(2) Determine the proportion of all cells that actually differentiate on any given day of the time course
Hypothesis	Early on, the curve will resemble an exponential growth curve with a plateau between d8-d9 and a decrease on d10	(3) Fit data to a curve to model differentiation
Possible problems and solutions	The curve may be defined by a complex equation or there may not be a peak on the curve—Step 1 is not useful, proceed to Step 2	(4) Use the curve and data from the preliminary step to estimate the peak day and standard deviation (SD) for differentiation of each cell fate

Step 2: Flow cytometry 2		Research goals

Markers used	All (Tables 1 and 3)	(1) Determine the peak day for differentiation of each cell type by cycling through iterations of data collection
Time course	Peak day ± SD as determined in step 1; 24 hour intervals for iteration 1; 12 hour intervals for iteration 2	After iteration 1, the time between intervals and the time course being analyzed will be narrowed, and the number of replicates conducted will be increased
Hypothesis	Peak day for each cell type will be different; endo = d3; ecto = d7; meso = d7	(2) Generate a comprehensive probabilistic model of ES cell differentiation over the course of time
Possible problems and solutions	Differentiation of germ layers can't be modeled—the experiment will be conducted with late markers of differentiated cell types	

Step 3: Flow cytometry and STAT3 function		Research goals

Markers Used	All (Tables 1 and 3); GFP ES cell line	(1) Use the model generated in Step 2 as a tool to analyze the effect of STAT3 on differentiation
Time course	Peak day ± SD from the model generated in Step 2	(2) Determine the peak day for differentiation of each cell type in cells that express dominant negative STAT3
Hypothesis	The absence of STAT3 will prevent differentiation of mesoderm and will delay or decrease ectoderm differentiation	(3) Use statistical analysis (repeated measure ANOVA) to determine whether the loss of STAT3 leads to a change in the progression of ES cell differentiation over time
Possible problems and solutions	STAT3 has no effect on differentiation—a new target for modification of differentiation will be chosen	(4) Confirm our results using GFP ES cell line and survival analysis
